# Analysis of Uncertainty and Variability in Finite Element Computational Models for Biomedical Engineering: Characterization and Propagation

**DOI:** 10.3389/fbioe.2016.00085

**Published:** 2016-11-07

**Authors:** Nerea Mangado, Gemma Piella, Jérôme Noailly, Jordi Pons-Prats, Miguel Ángel González Ballester

**Affiliations:** ^1^Simbiosys Group, Universitat Pompeu Fabra, Barcelona, Spain; ^2^International Center for Numerical Methods in Engineering (CIMNE), Barcelona, Spain; ^3^Catalan Institution for Research and Advanced Studies (ICREA), Barcelona, Spain

**Keywords:** uncertainty quantification, finite element models, random variables, intrusive and non-intrusive methods, sampling techniques, computational modeling

## Abstract

Computational modeling has become a powerful tool in biomedical engineering thanks to its potential to simulate coupled systems. However, real parameters are usually not accurately known, and variability is inherent in living organisms. To cope with this, probabilistic tools, statistical analysis and stochastic approaches have been used. This article aims to review the analysis of uncertainty and variability in the context of finite element modeling in biomedical engineering. Characterization techniques and propagation methods are presented, as well as examples of their applications in biomedical finite element simulations. Uncertainty propagation methods, both non-intrusive and intrusive, are described. Finally, pros and cons of the different approaches and their use in the scientific community are presented. This leads us to identify future directions for research and methodological development of uncertainty modeling in biomedical engineering.

## 1 Introduction

In the last decades, the finite element (FE) method has gained popularity within bioengineering problems, becoming a standard procedure in the implementation of biomedical systems. Most often, simulations only take into account predefined conditions, which consider a set of fixed values, carrying out a deterministic study. However, these conditions present uncertainties, which are omnipresent in all modeling approaches. In the bioengineering field, considering uncertainties has a high impact in the output due to their clinical relevance.

Uncertainty quantification is one main aspect of uncertainty management and is the name given to a set of techniques to deal with uncertainties. It consists of several aspects: (1) uncertainty identification, the first step to identify the source of variability uncertainty, (2) uncertainty categorization, which describes the kind of uncertainty regarding its source, (3) uncertainty characterization, which deals with the statistical description of the input, (4) uncertainty propagation, which analyzes the effects of the input variability on the output, and, finally, (5) uncertainty analysis, which assesses the variability effects and their sources.

Quantifying and dealing with such uncertainties has been explored in biomedical modeling and computational simulation to support diagnosis (González Ballester et al., 2000, 2002, 2004; Kohara et al., 2011; Schmid et al., 2011), pre and post-operative decisions (Talib et al., 2005; Rajamani et al., 2007; Bode et al., 2012), and therapy treatments (Belenguer et al., 2006; Zheng et al., 2007, 2009; Bednarz et al., 2011; Cao et al., 2012; Rüegsegger et al., 2012). Uncertainties influence in both the computation of the output and its interpretation. Thus, the aim of uncertainty quantification and propagation analysis is to determine the impact of uncertainty and variability on the response of the model. While probabilistic and non-probabilistic methods can be used to obtain this response, this article focuses mainly on probabilistic analysis. Applications of probabilistic analysis in bioengineering range from biomechanical analysis of implants across a given population (Belenguer et al., 2006; Kozic et al., 2010) to the study of morphometrical changes in body structures to diagnose diseases (Linguraru et al., 2006, 2008).

In computational modeling, uncertainty and variability can have many different sources. Some of them are related to the model itself, such as the ones that come from simplifications in the model, while others are related to the input parameters. There are also numerical approximation uncertainties that are related to the approximation of the numerical solution and model-form uncertainties that result from all the assumptions, abstractions, and mathematical formulation.

Uncertainty can be categorized into aleatory and epistemic. Aleatory uncertainty is related to the intrinsic variation of the system caused by model input parameters, which can lead to an unpredictable variation in the outcomes (Roy and Oberkampf, 2010; Lin et al., 2012). It can also be referred to as “variability,” “irreducible,” “stochastic,” or “random uncertainty” and is usually characterized using probabilistic approaches due to its random nature. In contrast, epistemic uncertainty stems from the lack of knowledge of the real system behavior. It is related to the approximation of the numerical solution. The error in these assumptions can be due to a wrong model interpretation or reductionist hypotheses of the governing equations. Theoretically, it can be overcome by defining a better physical-mathematical model or considering more data by carrying out thorough measurements. It is also known as “subjective” or “reducible uncertainty.” Epistemic uncertainty is not well characterized by probabilistic approaches, as it relates to the lack of knowledge, rather than statistical information (Lin et al., 2012).

**Figure 1:**
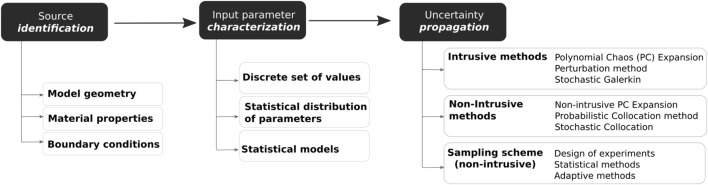
**General workflow analysis of input uncertainty and variability in computational models: (1) identification of all sources of uncertainty, (2) characterization of model input uncertainty, and (3) propagation of input uncertainty through the model**.

**Figure 2:**
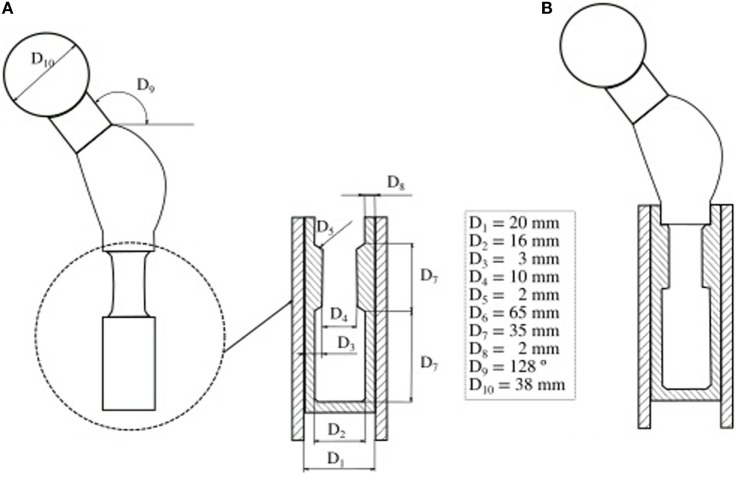
**Probabilistic design of a cemented hip prosthesis, adapted from (Kayabasi and Ekici, 2008)**. **(A)** Geometry of a hip prosthesis and variable design parameters. **(B)** New design parameters which reduce the probability of failure.

Classical probabilistic analysis predicts the output according to the uncertainty in input data, rather than from a predefined set of inputs like in deterministic studies. Uncertainty analysis using probabilistic descriptions of model inputs can be employed to describe the probabilistic distribution of the model outputs. For this, methods are needed to propagate the extensive range of uncertainties present in the model, such as those coming from the model geometry or material properties (Figure 1). These propagation methods can be divided into intrusive and non-intrusive (Lin et al., 2012). Intrusive models require reformulating the governing equation of the model, while non-intrusive methods use ensembles of simulations created by a sampling scheme. Non-intrusive methods are often preferred since commercial FE solvers can be used as black-boxes. On the other hand, intrusive approaches are usually in-house codes and need to be implemented according to specific conditions of the physical problem and constitutive model. Figure 2 gives an overview of the pipeline for the analysis of uncertainty and variability in the context of computational FE simulations.

This article reviews the methods and approaches to analyze input aleatory uncertainties in biomedical models for FE simulations. Characterization and propagation methods are presented in Sections 2 and 3, respectively. Sampling techniques and non-intrusive methods, such as random sampling and stochastic collocation, are discussed among others. Also, intrusive methods in the stochastic finite element framework are presented. Finally, Section 4 discusses current limitations and future perspectives for research.

## 2 Uncertainty and Variability in Computational Models: Identification and Characterization

The first step in the analysis of uncertainty is the identification and characterization of the different sources that can affect the outcome of the system. Such sources may have their origin in model inputs (Roy and Oberkampf, 2010), which include parameters such as material properties, geometry, and boundary conditions. Different techniques can be used to model input parameter variations. The simplest way consists in defining a set of values for each parameter, where no statistical distribution function is defined (section 2.2.1). Another approach is to consider a statistical distribution of the input parameters, such as a Gaussian or lognormal distributions, among others (section 2.2.2). Finally, a more accurate approach is to create statistical models where the whole variability across the selected population is considered (section 2.2.3).

### 2.1 Uncertainty Source Identification

Three main uncertainty sources that affect the inputs of computational models are: geometry, material properties, and boundary conditions. Most authors have focused on simplified FE models considering exclusively uncertainties in material properties and/or loading conditions (Nicolella et al., 2006; Easley et al., 2007; Dopico-González et al., 2009; Berthaume et al., 2012). However, others are aware that model geometry plays a key role in the behavior of anatomical structures, since morphometrical variations have important effects on the output results (Easley et al., 2007; Noailly et al., 2007; Kozic et al., 2010; Mousavi et al., 2012; Niemeyer et al., 2012; Rao et al., 2013). Considering different sources simultaneously allows coping with a wider range of uncertainties and hence provides a better accuracy in the model output.

### 2.2 Source Variability Characterization

#### 2.2.1 Discrete Sets of Values

The simplest approach to characterize input parameter variation is to use combinations from a predefined set of fixed values of the input data. The best way to do that is by using design of experiments (DOE) (section 3.1.1), which explore the simultaneous effect of multiple input variables (factors) on the output (response) (Yoon et al., 2005; Chen et al., 2011). DOE determines discrete values within a range for each factor, called levels. Assuming there are *f* factors, each one with *l* levels, the number of runs to be evaluated is equal to *l*^f^, increasing exponentially. Thus, depending on the amount of levels of each factor, the space of possible combinations varies. For instance, from 324 runs for the cervical cage evaluation (Yang et al., 2007) to 1024 for the pressure-relieving foot orthosis (Cheung and Zhang, 2008) for a full DOE.

The applications of these methods have not been extensively explored in bioengineering problems. Nonetheless, specific literature regarding product design and sensitivity analysis can be found. Malandrino et al. (2009) carried out a sensitivity analysis of an intervertebral disc FE model to analyze the influence of tissue material properties. Yang et al. (2007) used DOE to optimize a cervical ring cage by FE analysis and Cheung and Zhang (2008) a pressure-relieving foot orthosis. Both identified the sensitivity of the design factors and tested the effect of the structural combinations of FE models created by DOE.

Although DOE methods have been mainly used for product design optimization, they provide a simple approach to generate a group of instances that cover all combinations of parameters previously defined. Thus, the population of models created could be used also to account for the uncertainty derived by geometrical tolerances of a device or for anatomical variability, for instance, which is unfeasible to capture on a deterministic model.

#### 2.2.2 Statistical Distribution of Parameters

For source variability characterization, the most widespread approach considers a statistical distribution of the input random parameters (Easley et al., 2007). Biomechanical analyses that consider model input uncertainty using statistical distribution are usually focused on the study of joint behavior or prostheses. For instance, one can add information about the variability of geometrical parameters to analyze failure probability of a hip prosthesis (Bah and Browne, 2009), to validate probabilistic models (Mehrez and Browne, 2012), or to propose an optimized design (Kayabasi and Ekici, 2008) (Figure 2). The effects in a hip replacement in terms of strain fields generated by the combination of applied loads, bone and implant stiffness (Viceconti et al., 2006; Dopico-González et al., 2009), and bone-implant angle (Dopico-González et al., 2010a,b) have also been evaluated.

Other issues to take into account are the effect of the alignment variability (Laz et al., 2006a), loading profiles, friction, and wear coefficient (Laz et al., 2006b; Easley et al., 2007; Pal et al., 2008). To characterize the relevant parameters to be considered in a knee implant design, Fitzpatrick et al. (2010) identified the relationship between design parameters, surgical alignment, and loading conditions. Also accounting for component alignment variability, Rohlmann et al. (2009) found a strong correlation between alignment and gap size of an artificial disc and joint forces in the lumbar spine.

Although spine, knee or hip related studies are the most common ones, due to their clinical relevance, other studies can also be found. For example, related to hand representation, Valero-Cuevas et al. (2003) accounted for 50 musculoskeletal parameters to create a realistic model of the thumb. Berthaume et al. (2012) focused on a craniofacial FE model, where uncertainties in material properties were considered to determine the stress and strain output variability.

Some authors used also anthropological parameters, such as body segments, to perform inverse dynamic studies (Holden and Stanhope, 1998; Rao et al., 2006; Langenderfer et al., 2008). In a similar way, others have investigated the knee kinematic variability due to the uncertainty in anatomical landmark locations (Morton et al., 2007).

Other works involved the study of shear strength and fatigue of bone cement (Nicolella et al., 2006; Jeffers et al., 2007) or fracture risk prediction of the bone (Laz et al., 2007). In a more detailed way, Grasa et al. (2005) created a damage model for bone cement with the hip joint contact force as a random variable. Using this probabilistic damage model, Pérez et al. (2006) predicted the failure probability of the stem–cement interface accounting for the non-linearity of the cement degradation, rather than considering it linear (Grasa et al., 2005).

All these methods represented random parameters as Gaussian or lognormal distributions, defined by experimental results or from the literature. However, these variables or features could be misrepresented due to an imprecise characterization for not considering the dependency between them. Thus, more accurate models to represent the variability found in the population under study, as those described in the next section, are needed.

#### 2.2.3 Statistical Models

A more detailed approach to characterize source variability creates statistical models able to capture the variation of the input data. Based on Cootes’ theory (Cootes and Taylor, 1995), statistical shape models (SSM) are capable to capture the complex geometric variability of a large number of shapes within a class or population. Shapes are represented by a set of points called landmarks, forming a point distribution model. Through principal component analysis (PCA), the dimensionality of the object is reduced from a large set of correlated variables to a compact set of uncorrelated ones (Rajamani et al., 2007; Zheng et al., 2009, 2010; Baldwin et al., 2010; Bredbenner et al., 2010; Bonaretti et al., 2011; Fitzpatrick et al., 2011a). Then, the SSM provides the mean position of the points as well as the modes of variation that capture the main variability of the set of shapes. Further work adds gray-level intensity information to yield a so-called statistical appearance or density model (Cootes and Taylor, 2001). Then, gray-level value and density properties (e.g., of the bone tissue) can be related and the variability of the tissue material properties can be represented (Belenguer et al., 2006; Bryan et al., 2010; Bonaretti et al., 2011; Nicolella and Bredbenner, 2012).

**Figure 3:**
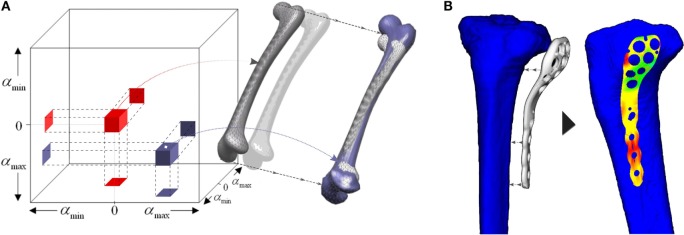
**(A)** Shape space defined by the PCA of a population of human femur. **(B)** Implant-fitting method applied for tibial orthopedic implants. Adapted from Kozic et al. (2010).

This approach has become a robust and powerful tool in bioengineering to extract the shape and density variability of the morphometrical data among patients. Researchers have applied SSM to a wide range of applications. Some studies focused on relating geometry to diseases, for instance the risk of developing osteoarthritis (Bredbenner et al., 2010) or geometry to kinematics (Smoger et al., 2015), creating thus a statistical shape–function model. Others considered the study of implant behavior or even optimization of implant designs across the population (see Figure 3) (Belenguer et al., 2006; Kozic et al., 2010). For instance, Belenguer et al. (Belenguer et al., 2006) presented a framework for orthopedic implant design using both statistical shape and intensity models. They aimed at finding the implant shape to fit the maximum percentage of patients across a given population. Also considering both shape and intensity statistical model, Nicolella and Bredbenner (2012) developed a parametric representation of the proximal femur to study how the structure affects bone strength.

The deformation behavior of an anatomical structure due to a surgical procedure or a medical test can be considered too. Ashraf et al. (2002) created a statistical estimation tool for intra-operative prostate deformation. The main modes of variation were extracted from the deformation of the prostate during transrectal ultrasound (TRUS) probe insertion, carried out computationally with different insertion parameters. Khalaji et al. (2008) estimated in real time the tissue deformation of prostate during TRUS by statistical models and neural networks. Similarly, Mousavi et al. (2012) compared multilayer neural networks and PCA to better fit the relationship between the prostate shape and FE deformation and stress fields.

The methods described above create a FE mesh for each sample of the point distribution model. Another approach is to employ a mesh-morphing framework (Bah et al., 2009), where a reference template mesh is morphed onto the remaining surfaces of the data set. In particular, to consider the material properties of the bone, Bryan et al. (2009) assigned to each node a value corresponding to the gray intensity extracted from a computer tomography scan. The statistical model was built by morphing a template tetrahedral mesh by elastic surface matching registration and mesh morphing (Bryan et al., 2010).

**Figure 4:**
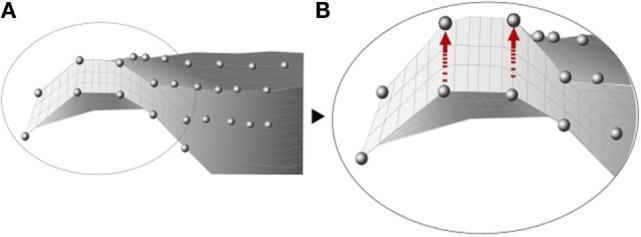
**Illustration of the internal nodes of a mesh before (A) and after (B) the morphing, adapted from Baldwin et al. (2010)**.

**Figure 5:**
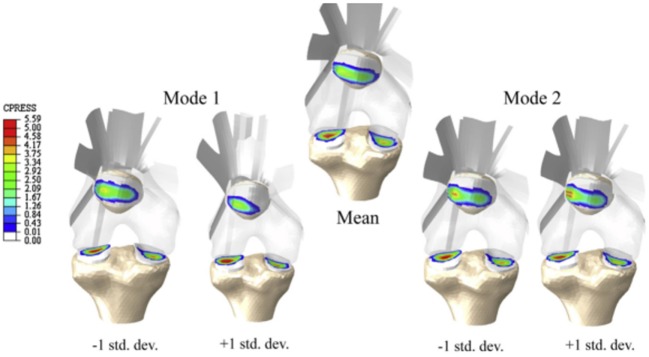
**Knee contact pressure (in MPa) of the statistical shape and alignment model (Rao et al., 2013)**. Models presented correspond to the mean shape and the variation of the first two modes between ±1 SD from the mean shape.

Baldwin et al. (2010) developed an integrated segmentation approach based on mesh morphing (Figure 4), where a template was morphed according to the manually manipulated perimeter of anatomical structures by a graphical user interface. Once the meshes from the subjects were created, a PCA was applied to capture the variability in the control points that define each structure of the joint, including size, shape changes, and positional alignment. Using this mesh-morphing approach, Fitzpatrick et al. (2011a,b) analyzed the changes in knee kinematics and contact mechanics due to articular alignment. These studies were followed by Rao et al. (2013) and Smoger et al. (2015) who evaluated and related knee mechanics and kinematics to both shape and alignment variability (Figure 5). Malandrino et al. (2015) used a similar approach to morph a generic mesh of lumbar vertebrae to subject-specific geometries and assess the interaction among biomechanical and biophysical processes and intervertebral disc condition. Despite the promising results obtained with these approaches, Bonaretti et al. (2011) found that a better mesh quality to generate a femur mesh – regarding the homogeneity of edge ratio and minimum angle – was obtained using image-based models, rather than by using a mesh-morphing approach to conduct a strain analysis at femoral neck. However, the FE results using both approaches slightly differed, and the choice of one specific solution is also often driven by the desired type of elements. Morphing-based methods have the advantage that they deform the same template mesh, which results in isotopological meshes and one to one correspondences. This means that the resulting meshes have the same number of nodes and elements and the same connectivity. This allows for an easier treatment in further steps of the computational FE analysis, such as boundary condition definition. On the other hand, large deformations of the reference mesh may lead to FE meshes with degenerated elements. In order to avoid this, quality checks are performed and fitting constraints are included in the morphing process, based on mesh quality measures such as the aspect ratio.

## 3 Uncertainty and Variability in Computational Models: Propagation

Uncertainty quantification of input parameters needs to be propagated through the simulation model to system outputs. Propagation methods can be divided into intrusive and non-intrusive (Figure 1). Intrusive methods reformulate the deterministic standard FE method in a stochastic FE method (SFEM) by integrating the random variables. Since the structural system is usually modeled by partial differential equations (PDE), the stochastic nature of the uncertain system is analogously modeled as a random field or stochastic process by stochastic PDE (SPDE). Despite its computational complexity, SFEM has received an important attention over the last decades, since it is a powerful tool for the solution of SPDE, and the growth of computational power makes it feasible to deal with large-scale problems. Non-intrusive techniques, rather than modifying the mathematical model, use sampling methods to generate an ensemble of simulations where each model is created by a sampling scheme accounting for model input variability. Then, each model is analyzed through FE and the results are finally statistically studied. In this review, pure sampling methods that create instances of the whole computational model have been classified into sampling techniques, including design of experiments, statistical methods and adaptive sampling methods. In biomedical computational models, non-intrusive methods are often preferred for practical reasons since, unlike intrusive methods, they do not require modification of the mathematical and numerical formulation of the underlying deterministic model. In this section, sampling methods are first described. Afterward, intrusive and non-intrusive methods are presented and discussed (see Table 1 for a comparison of some of their main characteristics).

**Table 1:** **Comparison of main uncertainty propagation techniques**.

	Statistical methods			Intrusive methods				Non-intrusive methods	
			
	DOE	MC	Multi-level MC	PCE	Perturbation method	Stochastic Galerkin	Fuzzy FE method	PCM	SCM
	
Runs	Fixed	Samples	Samples	Fixed and weighted	Fixed and weighted	Fixed and weighted	Fuzzy values	Fixed and weighted	Fixed and weighted
Statistics (*μ*, *σ*, Skew, Kurt)	All (low accuracy)	All	*μ*, *σ*	*μ*, *σ*	*μ*, *σ*	*μ*, *σ*	*μ*, *σ*	*μ*, *σ*	*μ*, *σ*
Number of uncertainties	Unlimited	Unlimited	Unlimited	Restricted due to computational cost	Restricted due to computational cost	Restricted due to computational cost	Restricted due to computational cost	Restricted due to computational cost	Restricted due to computational cost
Estimated calculation time	Depends on number of uncertain parameters	Expensive but constant	Cheaper than standard MC	Cheaper than the non-intrusive	Cheap	Cheaper than probabilistic collocation	Affordable	Exponentially increases with the number of uncertain parameters	Exponentially increases with the number of uncertain parameters
Statistical sampling	No	Yes	Yes	Yes	Yes	Yes	No	Yes	Yes

### 3.1 Sampling Techniques

Propagating uncertainty and variability can be easily done by means of sampling techniques such as statistical sampling methods or DOE. These methods are able to cope with a high number of computational simulations, where the results of the problem have to be obtained through FE analysis for each of the models created.

#### 3.1.1 Design of Experiments (DOE)

DOE is a statistical technique which studies the effect of multiple variables simultaneously defined as discrete sets of values (Yoon et al., 2005; Chen et al., 2011) (see 2.2.1). It received a special attention mainly in industries, such as electronics or chemistry, but it was recently adopted into bioengineering (Montgomery, 1997; Guvenis, 2013). In particular, some authors pointed out its potential in medical imaging (Taner and Sezen, 2007), and it was defined as a cost-effective way to analyze multiple parameters in biomedical research (Li, 1998).

In a classical DOE, one variable is changed randomly while keeping the others constant. However, this implies assessing a large number of combinations, and it is not easy to neither establish interaction between parameters nor determine the optimum. On the other hand, improved methods offer an optimized approach carrying out an efficient performance analysis where the number of simulations is minimum and the optimization process can include multiple objectives (Guvenis, 2013). In particular, the most widely used statistical design method is the Taguchi method (Dar et al., 2002). It is based on factorial analysis, in which only a fraction of all possible combinations of the experimental runs of a full factorial design. Thus, an efficient DOE is carried out with fewer simulations. In order to do that, factors are assigned to a crossed array layout. Controllable factors are part of the inner array, and uncontrollable ones are part of the outer array. Usually, the controllable factors are the ones that improve product characteristics (Yoon et al., 2005) and uncontrollable factors those that cause variations in a production process (Khuri and Mukhopadhyay, 2010). Once the value or level of the controllable factors is established, each run from the controllable factor array is tested across each run from the uncontrollable one. In other words, a series of experiments is carried out to obtain the optimal combination of parameters. This combination will have the greatest effect on the final performance with the minimum variation of the design (Yang et al., 2007; Cheung and Zhang, 2008). Finally, a statistical analysis can be performed to determine the contribution and significance of the main effects of each factor to conduct a sensitivity analysis or to assess the uncertain outcome for a given combination of parameters.

The strengths of the Taguchi method are (1) consistency and robustness of performance considering noise factors and (2) the reduction of time and cost of production (Yang et al., 2007). However, this method has also received criticisms due to both the large number of runs that are required and the difficulty to estimate interaction among control factors (Khuri and Mukhopadhyay, 2010). The Taguchi method has been successfully applied in bioengineering applications. For instance, studies of lumbar or cervical intervertebral discs (Espino et al., 2003; Ng et al., 2004; Malandrino et al., 2009, 2015), designs of a pressure-relieving foot orthosis (Cheung and Zhang, 2008), cervical ring cage (Yang et al., 2007), or even a finger probe for non-invasive hemoglobin monitor (Yoon et al., 2005).

Other approach related to DOE is the response surface method, also known as surrogate model or meta-model. However, this method is exclusively focused on design optimization. It consists to find the optimal parameters by iteratively fitting a mathematical model to the results obtained experimentally (Guvenis, 2013). A series of DOE are carried out to determine the optimum and the relationship between input and output results. For each settings of input variables, the response is measured. The fitting of these responses is known as the response surface (Khuri and Mukhopadhyay, 2010).

#### 3.1.2 Statistical Sampling Methods

Monte Carlo method is a statistical sampling technique in which a random value of each input variable is generated according to a prescribed probability density function. This method is very useful to compute full statistics, and it is considered an exact method to deal with uncertainty since it does not require any assumptions or approximations. However, the simulations computed must converge from a statistical point of view, which is one of the main drawbacks of the method. In computational bioengineering, Monte Carlo method has been applied to a large number of studies, such as modeling fatigue damage evolution in bone (Pidaparti et al., 2001) or the creation of a new hip prosthesis design (Kayabasi and Ekici, 2008). Specifically, Monte Carlo simulation has been used to evaluate artificial joint replacements (Kayabasi and Ekici, 2008; Bah and Browne, 2009; Rohlmann et al., 2009; Dopico-González et al., 2010b; Fitzpatrick et al., 2010, 2011a), to analyze the structural behavior of bone or biomaterials (Pidaparti et al., 2001; Jeffers et al., 2007), and to study anatomical structures (Valero-Cuevas et al., 2003; Belenguer et al., 2006; Bryan et al., 2009; Lin et al., 2009).

Latin Hypercube sampling can be classified as a stratified sampling method and provides an improvement of the convergence ratio over Monte Carlo simulation. It divides the sampling space into subsets – or equally probable intervals – and selects values from each of them randomly, rather than generating samples independently from the previously generated ones. This independence is one of its main advantages over Monte Carlo, since a fewer number of runs are needed to accurately approximate a random distribution (Laz and Browne, 2010; Lin et al., 2012). Latin Hypercube sampling is often used in uncertainty analysis, and, more specifically, it has been applied to study the design of mechanics of joint replacement (Chang et al., 2001; Bah and Browne, 2009; Dopico-González et al., 2009), the behavior of anatomical structures such as the craniofacial structures (Berthaume et al., 2012), the lumbar spine (Niemeyer et al., 2012) or the femur (Bonaretti et al., 2011), or even to analyze multivariable sensitivity in patelofemoral mechanics (Fitzpatrick et al., 2011a).

Most probable point-based (MPP) methods also present a better computational efficiency than Monte Carlo simulation since they reduce the analysis time and have a good accuracy–efficiency ratio (Easley et al., 2007). MPP methods represent the combination of parameters which predicts a certain performance within a specific probability level (Easley et al., 2007; Laz and Browne, 2010). Thus, they determine the most probable point of an objective function by applying an optimization scheme such as first-order Taylor series approximation. MPP methods are the basis of well-known reliability methods like first- and second-order reliability methods (FORM/SORM) (Zhao and Ono, 1999). These have been commonly applied in the structural analysis field to study the reliability of a system to estimate the mechanical failure probability. Although FORM has been rarely used in bioengineering, it has been employed to validate a model of a hip replacement as a time-efficient alternative to Monte Carlo (Mehrez and Browne, 2012).

The mean-value method obtains a mean-based response function and employs the MPP method. However, for higher orders, the advanced mean value (AMV) is used, which incorporates higher order terms to obtain a better representation of the response (Easley et al., 2007). Some authors have demonstrated in orthopedic and bone mechanics studies that the AMV is more efficient than the Monte Carlo simulation, reducing significantly the number of trials [e.g., from 1000 trials to 10 trials, approximately, in (Easley et al., 2007; Laz et al., 2007)].

Nicolella et al. (2006) found that a combination of Monte Carlo and MPP methods is more efficient than the individual use of either, although it requires a high number of runs to compute the entire response. They applied this combined probabilistic scheme to obtain the most influencial parameters in the design of a femoral hip prosthesis. AMV has been applied together with Monte Carlo in other applications such as kinematics and dynamics studies of the human body (Morton et al., 2007; Langenderfer et al., 2008).

#### 3.1.3 Adaptive Sampling Methods

Adaptive sampling methods perform a sampling strategy that chooses the next sample based on the results obtained by previous samples. These approaches usually focus on optimization since they are able to adjust the input parameters improving the results of the final model design. We have broadly divided them into two types: level sets and genetic algorithms.

**Figure 6:**
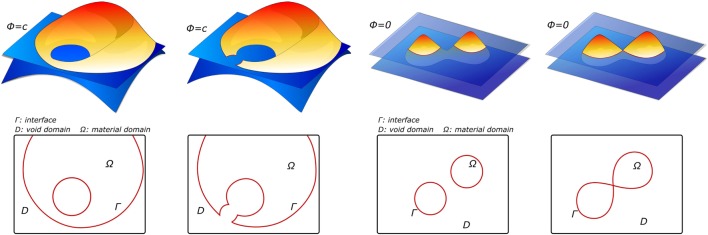
**Examples of level-set functions ϕ and their corresponding material domain Ω before and after the design update, adapted from Dijk et al. (2013)**.

Level sets have their origin in the image processing field. Specifically, they were employed as a segmentation technique based on active contours. Active contours, also known as snakes, are a technique based on evolving curves or surfaces that aims to detect boundaries of objects in an image, constrained by predefined geometrical conditions (Caselles et al., 1997; Chan and Vese, 2001). Level sets describe an interface Γ as a zero level of a continuous function *ϕ* defined on the image domain Ω. Thus, the continuous function ϕ is positive inside the domain, negative outside, and zero on the interface Γ (see Figure 6). This approach does not follow the interface itself, but it locates the set Γ(*t*) where the function ϕ vanishes (Osher and Fedkiw, 2001). Then, this makes it easier to follow interfaces with topology changes, such as holes or shape splits. For this reason, level sets are not only used as a segmentation method but also in other applications, such as topological optimization (Chen et al., 2010; Dijk et al., 2013) or tracking of moving objects (Paragios and Deriche, 2004). Level sets are widely used in medical image segmentation. In terms of physical interpretation as active contours, they are elastic bodies that react and move in a natural way to applied forces and constraints (McKeighen, 1996; Osher and Fedkiw, 2001).

Some authors adapted this approach of segmentation and tracking into a sampling method (Kozic et al., 2010). The authors used a level set-based optimization scheme in a shape space created by a PCA, thus creating a statistical shape model of human tibia. Rather than using level set as a shape representation method, they analyzed the statistical shape space to create a partition of the population, based on an implant-fitting criterion. This criterion was based on reducing the geometrical fitting error between the orthopedic implant, used for fracture fixation, and the surface of the proximal tibia (see Figure 3). Their scheme allowed them to find iteratively new samples in the shape space which meet certain geometry characteristics to finally obtain an implant design which satisfied the maximum percentage of the target population, according to a fitting criterion.

Another approach is based on the so-called genetic algorithms (GA). They are adaptive heuristic search algorithms rooted in the mechanism of evolution and natural selection found in genetics (Srinivas and Patnaik, 1994; Kumar et al., 2010). GA are based on a selection process which leads to the survival of the fittest individuals.

First, a set of initial instances, called population, are generated. Then, the population will be improved in successive iterations in the so-called generations. The set of parameters of a population are represented by a chromosome which will be evaluated assigning a fitness value. This value describes the measured solution according to an objective function and the results obtained. Thus, depending on their fitness value, some chromosomes are selected for the new generation. The higher the fitness value, the higher the probability of survival in the subsequent generation (Srinivas and Patnaik, 1994; Lodygowski et al., 2009; Kumar et al., 2010).

GA are particularly suited to find optimal solutions in complex problems. They have been applied in a wide range of fields such as biology, engineering, or computer science. Specifically, in bioengineering applications some examples are the optimization of prostate implants (Yu and Schell, 1996; Yang et al., 1998) or cochlear implant fitting (Choi et al., 2004; BaSkent et al., 2007; Legrand et al., 2007). Chang et al. (2015) used the idea of artificial neural network together with GA to optimize the screw orientation of an anterior cervical discectomy, obtaining an effective reduction of time and effort to find the optimal solution.

Despite the wide range of propagation methods presented so far, all of them are considered sampling schemes that eventually cope with a high number of computational models that need to be evaluated to obtain a statistically valid global response. In the next section, we present methods that deal directly with the uncertainty presented in the input random variables or fields. Both intrusive and non-intrusive methods can be used to propagate this input uncertainty. Some of the limitations of intrusive methods are the background required to implement them and their particularity (each modification of the physical problem involves defining again the SPDE), thus the implementation needs to be adapted to each physical problem addressed. Non-intrusive methods offer a good balance between generality and computational cost, being a common approach in engineering fields such as structural or aerodynamic studies, when uncertainty is considered.

### 3.2 Intrusive Methods

Intrusive approaches require to reformulate the deterministic standard FE method into a SFEM. Since the structural system is usually modeled by PDE, the stochastic nature of the uncertain system is analogously modeled as a stochastic process by SPDE. SFEM has received an important attention over the last decades as a powerful tool for the solutions of SPDE, thanks to the growth of the computational power, which makes it feasible and efficient to deal with these large-scale problems.

Intrusive methods consist in describing the uncertain input variable by a stochastic process or random field. Often, stochastic process denotes uncertainty on time, while random field refers to uncertainty on a domain in higher dimensions (e.g., space). They could be defined as families of random variables represented by their distribution and statistical moments. They may have a correlation function, or correlation length, that define the variability of the random field describing the dependency of the random variables in space (Keese, 2003). For continuous random variables, Gaussian random fields are often the most suitable and simplest, where all distributions are jointly Gaussian. However, non-Gaussian ones have also been employed lately due to the wide range of engineering problems with non-Gaussian characteristics (Sachdeva et al., 2007; Stefanou, 2009).

For numerical computation, random fields need to be discretized. This leads to treat both dimensions separately by defining two different meshes which are not required to be the same. The one to discretize the spatial dimension relies on the geometry, and it is usually created by standard techniques like FE. The second one is the “stochastic” mesh, used to discretize the stochastic dimension by approximating the continuous random field by a finite combination of random variables (Keese, 2003; Matthies and Keese, 2005; Stefanou, 2009).

Usually the FE mesh is coarser than the “stochastic” one. In contrast to the outcome seek through traditional mesh convergence studies, it has been even suggested that the later should be as fine as possible according to the computational resources available. To discretize the stochastic dimensions, different series representation techniques have been proposed, such as the mid-point method, the shape function method, or the optimal linear estimation (Keese, 2003; Sachdeva et al., 2007; Stefanou, 2009). However, the most common approach is the Karhunen–Loeve decomposition. Given the domain *D* and the sample space Ω, this approach, based on model reduction techniques, expands any random field, $k\left(x;\;\omega \right)\;:\;D\times \Omega \to R$, in a sum of products of functions of the stochastic and deterministic parameters in a Fourier-type series. In this way, a random variable is defined within the random field *k* at a certain point *x* ∈ *D*, thus a real number *k*(*x*, ω) is obtained for each realization *ω* ∈ Ω (Betz et al., 2004; Eiermann et al., 2007; Sachdeva et al., 2007).

(1) $$k\left(x;\omega \right)=\bar{k}\left(x\right)+\sum_{i=1}^{N}\sqrt{\lambda_{i}}k_{i}\left(x\right)\xi_{i}\left(\omega \right)$$

(2) ∫D Cov(x1,x2)ki(x1)dx1=λiki(x2)

where *ξ_i_* is a series of uncorrelated random variables, λ*_i_* and *k_i_* (***x***) are the eigenvalues and eigenfunctions of the eigenproblem [equation (2)], being *Cov*(*x*_1_, *x*_2_) the autocovariance function of the random field.

This approach is suitable for representation only when a few terms are needed to capture the maximum fluctuation of the random field that is defined by its correlation length. An upgrade of this technique is the proper generalized decomposition (Nadal et al., 2015), suitable to be used in any engineering and biomedical engineering problem. Another approach is the spectral representation method which similarly expands the random field as a sum of trigonometric functions with random phase angles and amplitudes (Eiermann et al., 2007; Stefanou, 2009).

Once the probability density function of the input random field is represented, it can be reformulated to introduce a finite dimensional subspace of the stochastic parameter space, *W*^h^ (see section 3.2.2) (Eiermann et al., 2007). This space, previously discretized, is created by a combination of the basis of the stochastic space. Several approaches have been proposed to construct this finite dimensional subspace. However, the most widely employed is the use of global polynomials in each random variable. This results on a space called polynomial chaos (PC) expansion.

#### 3.2.1 Polynomial Chaos Method

The PC method has been extensively used when dealing with uncertainty quantification in structural analysis and fluid dynamic studies. The method represents each random field *k* as an expansion of orthogonal polynomials, Φ*_i_*(*ε*(*ω*)), being *ε*(*ω*) and *ω* the random variable and the random results, respectively.

(3) $$k\left(x,t,\omega \right)=\sum_{i=0}^{N_{\mathrm{pc}}}k_{i}\left(x,t\right)\Phi_{i}\left(\varepsilon \left(\omega \right)\right)$$

(4) $$N_{\mathrm{pc}}+1=\frac{\left(n_{\mathrm{pc}}+p_{\mathrm{pc}}\right)!}{n_{\mathrm{pc}}!p_{\mathrm{pc}}!}$$

being *n_pc_* and *p_pc_* the dimensionality and order of expansion, respectively. This representation, treats independently the stochastic part, the polynomial chaos functions Φ*_i_*(*ε*(*ω*)), and the deterministic ones, the coefficients *k_i_* (*x,t*) (Loeven et al., 2008). When all parameters are independent Gaussian random variables, a basis of orthogonal polynomials sequence, called Hermite polynomials, is defined, creating the PC expansion space (Li and Zhang, 2007). Some issues, such as the convergence of the polynomial expansion, the high computational cost, and the complex implementation, have led to other approaches, such as non-intrusive PC method, to be able to use deterministic solvers for uncertainty studies (see section 3.3)

The solution of the stochastic response of the system already discretized is directly computed *via* high-dimensional integration over a probability space using different approaches (Fonseca et al., 2002; Eiermann et al., 2007; Sachdeva et al., 2007; Geneser et al., 2008; Stefanou, 2009). The most commonly employed is the direct numerical integration, which computes the solution in a straightforward way *via* Monte Carlo: *N* instances of the stochastic system matrix are sampled by Monte Carlo according to a probability measure (Matthies, 2008; Stefanou, 2009). Then, the system is solved *N* number of times and the population of response variability vector is computed by statistics (Stefanou, 2009). There exist a number of different numerical methods for solving the system of linear random equations, such as Monte Carlo (as previously mentioned), stochastic Galerkin approach, perturbation method, and Neumann series expansion, among others.

#### 3.2.2 Stochastic Galerkin Method

The Stochastic Galerkin method is a further development of the PC method used to approximate the stochastic response of the system *via* Galerkin methods (Pons-Prats, 2011). In the standard FE, the classical Galerkin method is a numerical technique to solve partial differential equations using a spatial discretization and weighted residual formulation to approximate the continuous problem to a discrete one. Analogously, the stochastic Galerkin method discretizes the random field using a PC expansion, achieving an efficient representation of the response with arbitrary probability density functions (Geneser et al., 2008).

This approach computes the stochastic process by a weighted sum of orthogonal polynomials of random variables, i.e., projecting the expansion of the random variables to the polynomials basis. Therefore, by using a stochastic projection Galerkin scheme, the solution lies in the stochastic subspace of order *m*. Hence, the approximation of the solution process can be defined as *u*(*θ*) = *ξ*_0_*ψ*_0_(*θ*) + *ξ*_1_*ψ*_1_(*θ*) + Λ *ξ_n_ψ_m_*(*θ*) = Ψ(*θ*)*ξ*, being $\Psi \left(\theta \right)=\left\{\psi_{1}\left(\theta \right),\ldots \psi_{m}\left(\theta \right)\right\}\in R^{n\times \left(m+1\right)}$ a set of basis vectors spanning the stochastic subspace *W*^h^ and $\xi ={\left\{\xi_{0},\xi_{1},...,\xi_{m}\right\}}^{T}\in R^{m+1}$ is a vector of undetermined coefficients (Sachdeva et al., 2007). By using this Galerkin projection scheme, the error of the expansion is minimized employing more basis vectors.

Once the approximation of the stochastic response is computed *via* stochastic Galerkin projection, the set of equations obtained for the expansion coefficients are deterministic, thus conventional quadrature methods can be used to evaluate the integral, and, therefore, the process is computationally cheaper than direct numerical integration (Xiu, 2007; Matthies, 2008).

#### 3.2.3 Perturbation Method

The perturbation method is another numerical technique to compute the solution of the stochastic process. It represents the output response of the system by expanding the output quantity around its mean value using Taylor’s series. Thus, it approximates the solution by a Taylor expansion with random coefficients (Lee and Lim, 1998). These coefficients are unknown deterministic vectors of the input uncertainty source (Kaminski, 2005; Stefanou, 2009), expressing in terms of lower-order polynomial function the stiffness matrix, load vector, and nodal displacement – in the particular case of a structural analysis – with respect to the random variables (Lee and Lim, 1998; Fonseca et al., 2002). The main drawback of this method compared to Monte Carlo approach, for instance, is its high intrusive character. There exists a high dependency between the evaluation of the sensitivity derivatives from the expansion and the code implementation.

**Figure 7:**
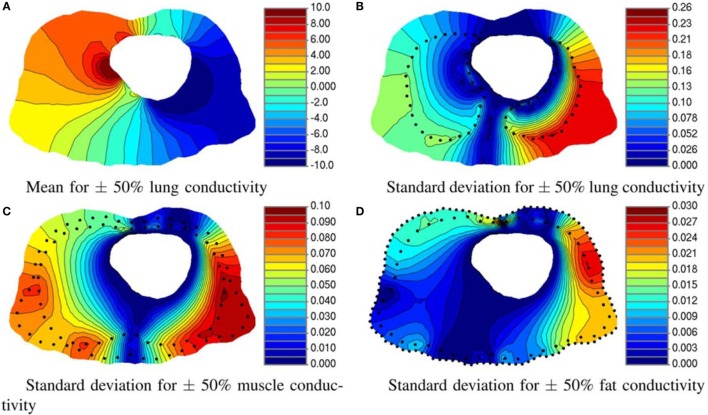
**Effect of tissue conductivity uncertainty of three organs on torso potential**. **(A)** Mean and **(B)** standard deviation of the electrical potential resulting from varying the lung conductivity. Standard deviation of the potential distribution on torso for 50% variation in **(C)** muscle and **(D)** fat conductivity. All units are in millivolts. (Geneser et al., 2008).

Instrusive methods have shown satisfactory results in only few applications in the bioengineering field. Regarding model reduction techniques, Karhunen–Loeve decomposition was used to simulate in real time the deformation of non-linear and anisotropic organic tissues, more specifically the human cornea, providing an alternative to FE analysis for the case study (Niroomandi et al., 2008). Geneser et al. (2008) presented a novel methodology which combines generalized PC expansion with stochastic Galerkin to study the sensitivity of organ tissue conductivity (see Figure 7). Shi and Liu (2003) estimated cardiac kinematic functions by SPDE. Finally, other authors compared different approaches, such as deterministic, probabilistic, and SFEM, in the study of a mid-cervical spine and concluded that SFEM could provide a high value to minimize uncertainty influences to carry out further reliability analysis of biological systems (Jang and Ekwaro-Osire, 2010).

The intrusive nature of these methods – high computational cost and extensive mathematical manipulation – still remains their main drawback. Some methods, such as non-intrusive PC expansion or the stochastic collocation method, were developed to address their limitations.

### 3.3 Non-Intrusive Methods

As mentioned, intrusive methods need to modify the governing equations, which could be difficult or even unfeasible. This led to the development of similar approaches, so as to facilitate the implementation of the analysis.

#### 3.3.1 Polynomial Chaos Method (Non-Intrusive Approach)

One of these approaches is the non-intrusive PC expansion. It estimates the coefficients for known orthogonal polynomial functions based on a response metric of interest according to a set of simulations. Thus, it creates a meta-model by encompassing the results space (Huberts et al., 2015). The response coefficients of the expansion can be computed by (1) spectral projection, which employs inner products and polynomial orthogonality properties, or (2) linear regression, called also point collocation, which extracts the coefficients that best match a set of output spaces by linear least square (Xiu, 2007; Eldred and Burkardt, 2009; Huberts et al., 2015). In order to estimate its coefficients, spectral projection can use a simple sampling approach – known as non-intrusive spectral projection (Loeven et al., 2008; Abgrall et al., 2010). The idea is to sample a discrete parameter space where the deterministic simulation is computed obtaining a set of responses (Abgrall et al., 2010), and the variable solution is reconstructed as a polynomial expansion. Huberts et al. (2015) reported a better performance of the least-squares regression approach, compared to the spectral projection, in a cardiovascular pulse wave propagation model (Huberts et al., 2015). PC expansion can be also employed together with surrogate models, previously introduced as response surface method, to reduce the computational cost, for instance, creating the surrogate model in the form of the Hermite polynomials (Isukapalli et al., 1998). Thus, the solution can be computed by fast-converging polynomial approximation based on least-squares linear regression (Du and Chen, 2000; Berveiller et al., 2006; Baroth et al., 2007; Eck et al., 2015).

#### 3.3.2 Probabilistic Collocation

The second technique to compute the coefficients of the expansion is the probabilistic collocation method. This approach combines the idea of PC expansions and the collocation approach. It represents the behavior of the uncertain parameters by a set of weighted values computed by a numerical integration, such as Gauss-quadrature, of the stochastic space based on Lagrange polynomials (Loeven and Bijl, 2008; Eldred and Burkardt, 2009; Pons-Prats, 2011). As mentioned previously, it uses the concept of collocation points to obtain the coefficients of the expansion that better represent the uncertain response (Eldred and Burkardt, 2009) by doing the Galerkin projection (Loeven and Bijl, 2008). Then, the approximation of the distribution function is integrated from each collocation point (Loeven and Bijl, 2008; Loeven et al., 2008; Eldred and Burkardt, 2009). The stochastic representation of a function is described as follows:

(5) $$f\left(x,\omega \right)\approx \sum_{i=1}^{N_{p}}f_{i}\left(x\right)L_{i}\left(\varepsilon \left(\omega \right)\right)$$

being *L_i_*(*ε*(*ω*)) the Lagrangian polynomial which leads to the calculation of the weight, *w_i_*, for each *f_i_*(*x*). It has been proved that this probabilistic collocation method is more efficient than the Galerkin PC expansion approach (section 3.2) and that the computational cost is reduced considerably compared to the direct integration method using Monte Carlo (Li and Zhang, 2007).

#### 3.3.3 Stochastic Collocation Method

Closely related to PC expansion, stochastic collocation is another non-intrusive method where the simulations are performed at specific collocation points in the uncertainty space, combining the fast convergence of the PC approach and the decoupled nature of sampling techniques such as Monte Carlo (Xiu, 2007; Loeven et al., 2008; Eldred and Burkardt, 2009; Pons-Prats, 2011; Sankaran and Marsden, 2011). The solution of the stochastic response is constructed employing Lagrange interpolation to obtain the expansion polynomial which is obtained using a collocation grid of points rather than by random sampling (Loeven et al., 2008; Eldred and Burkardt, 2009).

As described by Loeven et al. (2008), the solution in a stochastic domain *α*, which is defined according to a standard domain of orthogonal polynomials (Abgrall et al., 2010), is *u*(*x,t*,*α*). From the *α* domain, *N_p_* collocation points *α_i_* are taken. The solution *u*(*x,t*,*α*) is approximated by the following expansion

(6) $$u\left(x,t,\alpha \right)\approx \sum_{i=1}^{N_{p}}u_{i}\left(x,t\right)h_{i}\left(\alpha \right)$$

where *u_i_* (*x,t*) are the values of *u*(*x,t*,*α*) at the given collocation points *α_i_*, being *h_i_*(*α*) the interpolating polynomials of degree *N_p_* − 1 (Loeven et al., 2008).

This method provides a set of deterministic equations that are computed using standard solvers. That means that for each collocation point, a deterministic problem is solved. The main difference, compared to PC expansion is that it forms multidimensional interpolants for known coefficients, rather than estimating the coefficients for known basis functions of the orthogonal polynomial (Eldred and Burkardt, 2009).

These non-intrusive approaches have been employed also in the biomedical engineering fields, mainly in topics such as drug infusion (Preston et al., 2009), hemodynamics (Xiu and Sherwin, 2007; Sankaran and Marsden, 2011), or cardiovascular simulations (Eck et al., 2015, 2016). Both Sankaran and Marsden (2011) and Eck et al. (2015) employed the approach of stochastic collocation to carry out sensitivity analysis on blood flow simulations.

Concretely, Eck et al. (2015) evaluated how arterial stiffness influenced on backward-propagating pressure waves, using a previously developed model that employs PC expansion in order to set the parameters of the model to create a patient-specific predictive model (Huberts et al., 2015). Later, Eck et al. (2016) provided a guideline for the uncertainty analysis in cardiovascular applications. Sankaran and Marsden (2011) proposed an adaptive collocation algorithm to reduce the computational cost of the stochastic collocation scheme and quantify the confidence of the stochastic response in abdominal aortic aneurysm and carotid artery bifurcation simulations.

## 4 Conclusion

This article reviews the analysis of uncertainty and variability in the context of bioengineering computational models. Characterization techniques and propagation methods have been presented, as well as examples of their applications in biomedical simulations.

As shown, a great number of uncertainty and variability factors are found in bioengineering computational models. Among them, the most studied are the ones related to anatomical shape and implant design parameters. The techniques involving statistical shape models are the most commonly used due to their ability to capture the huge variability among patients and then obtaining realistic results according to the population.

The most common techniques of uncertainty propagation employed in this field are the ones within the sampling techniques category. These methods have the advantage that they do not require modifications to existing computational tools, hence the solver of FE can be used as a “black box,” and their use and implementation is straightforward. Even so, their computational cost is rather high due to the number of samples that need to be analyzed. Table 2 summarizes the studies within biomedical engineering field presented, where geometrical variability and non-intrusive approaches are the ones most considered.

**Table 2:** **Uncertainty studies according to the source of uncertainty and its characterization in the context of biomedical computational analysis**.

Characterization	Reference	Source			Propagation method
		Geometry	Material properties	Boundary conditions	
No statistical distribution	Yoon et al. (2005)	Photodetector size		Color LED light	Taguchi
	Yang et al. (2007)	Design parameters of cervical ring cage	Cervical ring cage		Taguchi
	Cheung and Zhang (2008)	Design parameters of relieving foot orthosis			Taguchi
	Bah et al. (2009)	Hip replacement rotation			–
	Malandrino et al. (2009)		Intervertebral disc		Factorial Anal.
	Ng et al. (2004)		Cervical spine		Factorial Anal.
	Espino et al. (2003)	Intervertebral disc anatomy	Intervertebral disc	Compressive force	Factorial Anal.
		Intervert. disc anatomy (gaus. distr.)	Interver. Disc (gauss. distr.)		MC
Statistical distribution	Easley et al. (2007)	Design parameters of hip stem	Hip stem		MC + MPP
		Knee replacement alignment		Load and coeff. of friction	
	Kayabasi and Ekici (2008)		Bone, cement, and prosthesis	Joint and muscle load	MC
	Mehrez and Browne (2012)	Bone radius	Bone	Joint load	MC + FORM
	Bah and Browne (2009)	Shape parameters bone and hip stem	Bone and hip stem	Joint load	MC + LHS
	Dopico-González et al. (2009)		Bone and hip replacement	Joint and applied load	MC + LHS
	Nicolella et al. (2006)		Bone and bone cement	Joint load	MC + MPP
	Laz et al. (2006b)	Knee replacement alignment		Load and coeff. of friction	MC + AMV
	Laz et al. (2007)		Bone		MC + AMV
	Fitzpatrick et al. (2011a) and Fitzpatrick et al. (2011b)	Implanted patellofemoral alignment		Muscle load	MC + LHS
	Fitzpatrick et al. (2010)	Surgical alignment		Joint load profile	MC
		Knee implant design (no stat. distribution)			
	Dopico-González et al. (2010a)	Hip prosthesis alignment		Contact force	MC
	Viceconti et al. (2006)	Implant size (no stat. distribution)	Bone	Body weight	MC
	Valero-Cuevas et al. (2003)			Musculoskeletal parameters	MC
	Berthaume et al. (2012)		Bone		MC + LHS
	Niemeyer et al. (2012)	Lumbar spine geometry			LHS
	Jeffers et al. (2007)	Spatial distribution of material pores			MC
	Rohlmann et al. (2009)	Artificial disc shape and alignment		Scar tissue	MC
	Langenderfer et al. (2008)	Body segments and anatomical landmarks			MC + AMV
	Morton et al. (2007)	Anatomical landmarks location			MC + AMV
	Rao et al. (2006)	Body segments			–
	Holden and Stanhope (1998)	Knee center location			–
	Geneser et al. (2008)		Organ tissue conductivity		PCE + SG
	Sankaran and Marsden (2011)	Carotid artery radius		Inlet velocity and flow-split	SC
		Abdominal aortic aneurysm size			SC
	Eck et al. (2015)		Arterial stiffness		SC
	Xiu (2007)	Arterial cross-section	Arterial stiff., blood density	Internal pressure and friction	gPCE
	Huberts et al. (2015)	Arterial/venous anatomy	Arterial/venous parameters	Cardiovascular pressures	gPCE
Statistical models	Bredbenner et al. (2010)	Knee subchondral bone surface			SSM
	Belenguer et al. (2006)	Femur geometry	Bone density		SSDM
	Kozic et al. (2010)	Proximal human tibia shape			SSM
	Ashraf et al. (2002)	Prostate shape			SSM
	Mousavi et al. (2012)	Prostate shape			SSM
	Bryan et al. (2009) and Bryan et al. (2010)	Femur geometry	Bone density		SSDM
	Baldwin et al. (2010)	Knee joint			SSM
	Fitzpatrick et al. (2011b)	Articular cartilage geometry			SSM
	Rao et al. (2013)	Knee joint (shape and alignment)			SSM
	Nicolella and Bredbenner (2012)	Femur geometry	Bone density		SSDM

*MC, Monte Carlo; MPP, most probable point; (A)MV, (advanced) mean value; FORM, first order reliability method; LHS, latin hypercube sampling; SS(D)M, statistical shape (and density) model; (g)PCE, (generalized) polynomial chaos expansion; SG, stochastic Galerkin; SC, stochastic collocation*.

Many methods, intrusive and non-intrusive, firmly settled into some engineering fields such as structural or fluid dynamic analysis, have become regular tools to study uncertainty and variability in computational models. However, intrusive methods have been rarely used in biomedical computational models. Intrusive approaches require a high knowledge of the computational problem since an adaptation of the deterministic case is needed. Despite their advantages, these approaches are not commonly used due to their high computational cost, issues related with the convergence of the polynomial expansions, and the high mathematical manipulation. In consequence, this led to study further non-intrusive methods, such as the non-intrusive PC method or probabilistic collocation (see Table 1 for a comparison of the main techniques discussed). These non-intrusive methods started to be used in the last decades, showing promising results in biomedical engineering, mainly in topics related with cardiovascular applications.

Furthermore, the different approaches discussed have been compared to each other in extremely few cases. It could be interesting to contrast the results obtained by a non-intrusive approach to an intrusive one in different applications to determine the feasibility of certain methods in biomedical fields. Another consideration to point out is that, in biomedical engineering, epistemic uncertainties are present too. Moreover, further uncertainties can be found in the data acquisition process. There is a lack of studies which consider both uncertainties, aleatory and epistemic, in the FE simulations. Considering both types of uncertainties would provide more accurate results according to data origin, methodology of processing, and the variability and uncertainty found in the physical problem definition.

Finding the best suitable method is a trade-off among several factors. Namely, the cost of implementing the method for each application – leading to the selection of an intrusive or a non-intrusive method, the computational cost associated with the specific analysis – leading to the selection of a low number of samples, or of an intrusive method – and many other considerations which are problem specific. The analysis with uncertainties is becoming more and more relevant in any engineering field, and more specifically in biomedical engineering. While the cost is still higher than a standard deterministic analysis, the benefits of better fitting the real world applications are a clear advantage.

From this review, it is clear that there is still work that can be done in bioengineering applications with respect to uncertainty quantification. Some of them would be, for instance: (1) carefully analyze and model the input parameters uncertainty, dealing not exclusively with geometrical parameters or material properties, but having also into account boundary conditions and altogether. It is a common problem in many engineering fields, but it takes a relevant importance when focusing on biomedical engineering problems due to its clinical relevance. (2) As mentioned, compare the efficiency and applicability of each method, both intrusive and non-intrusive. The simplicity of this fact could seem meaningless, but it can provide really useful information to choose the approach that better fit the study requirements, regarding computational cost and accuracy of the solution. Furthermore, the analysis between approaches should be done on the application under study, since the reference is the equivalent result on the deterministic approach, and the efficiency is directly related to each application. (3) Finally, the analysis of the output behavior is one of the key factors to take real benefit of uncertainty quantification. Finding the best statistical moments and parameters to represent the propagated variability, while obtaining a good understanding of the interaction between input–output variability efficiency is the final aim of uncertainty propagation methods.

The authors are aware that uncertainty quantification is a vast field, and some methods are missing in this review. Only those which have been already used in bioengineering applications have been studied, leaving other methods, such as fuzzy FE methods or multi-level Monte Carlo, for future reviews and applications.

## Author Contributions

All authors have contributed equally to this work and approved it for publication.

## Conflict of Interest Statement

The authors declare that the research was conducted in the absence of any commercial or financial relationships that could be construed as a potential conflict of interest.
